# Supplementary material for: Beta-Blocker Separation on Phosphodiester Stationary Phases—The Application of Intelligent Peak Deconvolution Analysis
Source: Molecules. 2023 Apr 5;28(7):3249. doi: 10.3390/molecules28073249 (PMC10096687; doi:10.3390/molecules28073249)
Supplement: Supplementary file 1 [file molecules-28-03249-s001.zip › molecules-2305672-supplementary.pdf]

Table S1 Retention factors of beta-blockers on tested phosphodiester statioanry phases.

Diol-P-Benzyl

ACN	metoprolol	propranolol	mexiletine	cicloprolol	atenolol	acebutolol	oxprenolol	pindolol
10%	14.68	13.87	8.37	16.99	10.05	15.60	13.38	8.98
20%	8.03	7.66	5.32	7.46	6.44	8.00	7.32	6.18
30%	6.25	5.62	4.80	5.80	6.02	6.20	5.50	5.05
70%	5.10	3.62	3.60	3.96	9.15	5.62	3.85	4.02
80%	7.12	5.24	4.95	6.07	16.67	8.83	5.75	5.96
90%	23.88	17.71	11.76	21.14	56.73	31.34	18.77	19.04

Diol-P-C18

ACN	metoprolol	propranolol	mexiletine	cicloprolol	atenolol	acebutolol	oxprenolol	pindolol
10%	18.96	20.94	11.43	16.90	8.24	26.08	21.89	11.00
20%	9.71	10.05	7.84	9.07	5.35	10.42	11.22	7.12
30%	6.76	5.47	5.99	5.84	4.57	6.71	7.45	5.51
70%	4.79	4.13	3.72	5.10	6.71	5.13	4.49	3.90
80%	6.80	5.44	4.86	5.69	11.89	7.64	5.55	5.51
90%	19.65	15.65	11.35	17.76	42.33	25.52	19.72	16.16

Diol-P-C10

ACN	metoprolol	propranolol	mexiletine	cicloprolol	atenolol	acebutolol	oxprenolol	pindolol
10%	7.28	9.98	4.39	9.57	4.44	9.34	6.94	5.00
20%	5.04	7.03	3.87	6.02	3.43	5.62	4.48	4.02
30%	4.07	5.17	3.44	4.60	3.05	4.25	4.20	3.51
70%	3.81	3.25	2.97	3.33	5.41	4.16	2.79	3.17
80%	4.81	4.62	4.07	4.18	9.08	6.33	4.09	4.17
90%	14.75	13.90	9.88	15.70	36.54	22.08	17.37	14.48

Diol-P-Chol

ACN	metoprolol	propranolol	mexiletine	cicloprolol	atenolol	acebutolol	oxprenolol	pindolol
10%	25.14	47.09	14.14	25.26	12.64	39.09	26.63	11.59
20%	12.89	22.38	9.56	13.40	8.38	15.45	13.74	7.82
30%	8.88	12.73	7.14	8.19	6.36	9.47	9.10	5.90
70%	5.65	4.85	4.30	4.24	8.49	6.25	4.67	4.56
80%	7.12	6.34	5.36	6.88	12.78	9.31	7.91	5.75
90%	21.87	19.59	13.71	22.94	49.06	32.66	25.25	18.23
